# The Influence of Flanking Secondary Structures on Amino Acid Content and Typical Lengths of 3/10 Helices

**DOI:** 10.1155/2014/360230

**Published:** 2014-10-13

**Authors:** Vladislav Victorovich Khrustalev, Eugene Victorovich Barkovsky, Tatyana Aleksandrovna Khrustaleva

**Affiliations:** ^1^Department of General Chemistry, Belarusian State Medical University, Dzerzinskogo 83, 220116 Minsk, Belarus; ^2^Laboratory of Cellular Technologies, Institute of Physiology, The National Academy of Sciences of Belarus, Academicheskaya 28, 220072 Minsk, Belarus

## Abstract

We used 3D structures of a highly redundant set of bacterial proteins encoded by genes of high, average, and low GC-content. Four types of connecting bridges—regions situated between any of two major elements of secondary structure (alpha helices and beta strands)—containing a pure random coil were compared with connecting bridges containing 3/10 helices. We included discovered trends in the original “VVTAK Connecting Bridges” algorithm, which is able to predict more probable conformation for a given connecting bridge. The highest number of significant differences in amino acid usage was found between 3/10 helices containing bridges connecting two beta strands (they have increased Phe, Tyr, Met, Ile, Leu, Val, and His usages but decreased usages of Asp, Asn, Gly, and Pro) and those without 3/10 helices. The typical (most common) length of 3/10 helices situated between two beta strands and between beta strand and alpha helix is equal to 5 amino acid residues. The preferred length of 3/10 helices situated between alpha helix and beta strand is equal to 3 residues. For 3/10 helices situated between two alpha helices, both lengths (3 and 5 amino acid residues) are typical.

## 1. Introduction

Although 3/10 helices play important roles in the folding of proteins (as parts of transmembrane helices, as interactive interfaces, as immunogenic epitopes, and as fragments of active centers), historically they were thought to be unstable and relatively rare [1, 2]. Indeed, there are many cases when a 3/10 helix is present in one 3D structure from Protein Data Bank (http://www.pdb.org/) and absent in another 3D structure of the same 100% identical protein. Ligand binding, changes in pH, interactions with other proteins, and other specific conditions may influence distances between nitrogen and oxygen atoms from the protein backbone and angles between N–H and C=O groups. Some of those changes lead to the appearance or disappearance of certain hydrogen bonds, frequently making N- and C-termini of beta strands and alpha helices longer or shorter. Because of the short length, 3/10 helices are prone to appearing or disappearing completely due to the formation or destruction of a single hydrogen bond. Even relatively small changes in distances between atoms may lead to transitions from alpha helix to 3/10 helix and vice versa. Those structural transitions are well studied in relatively short model peptides [3–5]. Even one of the newest methods for secondary structure prediction (C8-SCORPION) has an accuracy of 50% for 3/10 helices prediction when a template of 40% or more identity is available [6]. The authors of the “C8-SCORPION” state that such accuracy (50%) is a good result for 3/10 helices prediction [6].

According to the results of several studies [7, 8], the number of amino acid residues in 3/10 helices is about 10 times lower than the number of residues in alpha helices. However, that ratio between the number of residues in 3/10 helices and the number of residues in alpha helices cannot be considered as an argument towards justifying the extreme rarity of 3/10 helices. In fact, 3/10 helices are just about three times less abundant as elements of secondary structure than alpha helices. This situation can be easily explained by the low average length of 3/10 helices.

Most sources [1, 4, 9] refer to the same number reflecting the mean length of 3/10 helices (3.3 residues). That number has been revised in the present study. In fact, there are two common lengths of 3/10 helices: 3 amino acid residues and 5 amino acid residues. In our data set 3/10 helices containing 5 amino acids are even more abundant than those containing 3 residues. Both previous results [1, 4, 9] and the results of the present study are based on secondary structure assignment by the most widely used DSSP program [10]. According to the DSSP, the shortest possible 3/10 helix is the combination of two consecutive (*i*–*i* + 3) turns.

Yet another popular theory states that 3/10 helices are usually situated before or after alpha helices [1, 4, 9]. Moreover, those short helical fragments are thought to be able to undergo transitions from 3/10 helix to alpha helix and vice versa [4, 5, 8]. In our data set, the number of 3/10 helices situated near beta strands (after or before them) is almost the same as the number of 3/10 helices situated near alpha helices. Cases when 3/10 helix is situated between two beta strands are relatively frequent according to the results of previous works as well [9, 11]. This could show that 3/10 helices may not always be just an extension of an alpha helix.

It has been shown that random coil regions can be considered to function as “connecting bridges” between major elements of secondary structure (alpha helices and beta strands) [12]. Amino acid content of those coil regions strongly depends on their flanking structures. For example, coil between beta strand and alpha helix (BCH) is enriched by Ser and Thr, while coil between alpha helix and beta strand (HCB) is enriched by Arg and Lys. Coil between two beta strands (BCB) is enriched by Gly, while coil between two alpha helices (HCH) is enriched by Leu. The usage of Pro in HCH and HCB regions is higher than that in BCB and BCH ones [12].

The aim of the present study was to compare 3/10 helices with random coil structures. Since amino acid content of random coil depends on the flanking structures, we performed that comparison separately for four groups of connecting bridges including and not including 3/10 helices. That kind of comparison is adjusted by extremely short length (and often by the absence) of coil regions between 3/10 helices and “major” elements of secondary structure.

The present work showed that connecting bridges containing 3/10 helices in general follow some sharp trends that are known for “pure” coil regions [12]. This finding allows us to say that 3/10 helices may be described as parts of connecting bridges between “major” secondary structure elements which demonstrate some regular hydrogen bonding.

All the differences between connecting bridges with and without 3/10 helices were factored into probability scales used by the “VVTAK Connecting Bridges” algorithm (http://chemres.bsmu.by/). This algorithm shows what state (pure random coil or random coil with 3/10 helix) is more probable for a given connecting bridge.

The main issue focused on by this study is the nature of 3/10 helices. Are they nothing but random coil with two occasionally overlapping instable “*i*–*i* + 3” “main chain-main chain” hydrogen bonds, or are they short alpha helices (or even their parts) with different pattern of hydrogen bonding? Actually, there are two answers to that question. 3/10 helices composed of 3 amino acids can be thought of as products of a random coil, while 3/10 helices composed of 5 amino acids demonstrate many similarities to the N-termini (and not C-termini) of alpha helices. N-termini of alpha helices themselves are enriched by amino acids widespread in random coil [13, 14]. So, in our opinion, 3/10 helices are much closer to random coils than to alpha helices.

## 2. Materials and Methods

Amino acid usage in proteins depends on the GC-content of corresponding genes [15]. Such amino acids as Gly, Ala, Arg, and Pro (GARP) have higher levels of usage in proteins encoded by GC-rich genes and lower levels of usage in proteins encoded by GC-poor genes. In contrast, Phe, Tyr, Met, Ile, Asn, and Lys (especially, Ile, Asn, and Lys) have higher levels of usage in proteins encoded by GC-poor genes and lower levels of usage in proteins encoded by GC-rich genes [15, 16]. Natural selection on amino acid substitutions caused by mutational AT-pressure leads to the growth of A + G in AT-rich genes [17]. Proteins encoded by genes of different GC-content (and different AG-content) should be present in data sets used for amino acid content analyses of secondary structure elements, as well as in other studies on computational proteomics, in order to avoid biases caused by underrepresentation or overrepresentation of certain amino acid residues [18].

As a material for this work we used 542 3D structures of proteins from the Protein Data Bank (http://www.pdb.org/) annotated with the help of DSSP program [10]. Those proteins belong to twelve bacterial species. Four of those bacterial species have genomic GC-content lower than 40% (*Borrelia burgdorferi*,* Clostridium perfringens*,* Francisella tularensis*, and* Staphylococcus aureus*). Four species have genomic G + C levels between 40% and 60% (*Synechococcus elongatus*,* Porphyromonas gingivalis*,* Shigella flexneri*, and* Yersinia pestis*). There were also four bacterial species with GC-content higher than 60% (*Mycobacterium tuberculosis*,* Rhodococcus jostii*,* Xanthomonas campestris*, and* Streptomyces coelicolor*). PDB access numbers can be found in the Supplementary Material “PDBIDS” file (available online at http://dx.doi.org/10.1155/2014/360230).

Amino acid sequences of proteins used in this study demonstrate a level of similarity lower than 25% according to the results of the “Decrease Redundancy” algorithm (http://web.expasy.org/decrease_redundancy/). Therefore there were no homologues in our data set.

Less than half of the proteins (43.5%) used in this study have a record of classification by CATH (Class, Architecture, Topology, Homologous superfamily) system in PDB. Of those that had such records, there were 53.9% Alpha Beta proteins, 24.8% Mainly Alpha, 6.6% Mainly Beta, and 14.7% mixed proteins. Just 18.4% of proteins were already classified by SCOP (Structural Classification of Proteins) system. Among them there are 34.9% of “all alpha” proteins, 25.7% of “alpha and beta proteins (a + b),” 18.3% of “alpha and beta proteins (a/b),” 12.8% of mixed proteins, and 8.2% of “all beta” proteins. Together “a + b” and “a/b” proteins make 44.0% of the data set. So, according to both classifications, the most of the proteins from this study contain both alpha helices and beta strands.

We separated four types of connecting bridges, with and without 3/10 helices, from all of the analyzed proteins. Those regions are abbreviated according to the flanking “major” secondary structure elements (“H” is alpha helix and “B” is beta strand). There were 308 BIB, 240 HIH, 284 BIH, and 225 HIB regions. The letter “I” is used in this work to refer to the connecting bridge between “major” secondary structure elements containing 3/10 helix. Amino acid content of those four types of regions of coil containing 3/10 helices was compared with amino acid content of corresponding connecting bridges with “pure” random coil (designated by letter “C”): 2230 BCB, 1207 HCH, 1418 BCH, and 1681 HCB regions. We also compared amino acid content of BIB, HIH, BIH, and HIB regions with each other.

Probability scales have been created from amino acid usages for eight types of connecting bridges studied. Those scales have been incorporated into the “VVTAK Connecting Bridges” algorithm (they can be found in “scales” list). The “VVTAK Connecting Bridges” algorithm can be downloaded for free from the web page http://chemres.bsmu.by/. The algorithm was built via MS Excel spreadsheet. To utilize, one has to first copy FASTA protein sequence into the “sequence” list and copy the text from PDB file into the “PDB” list. As an output (on its “output” page) that algorithm provides information on expected conformational state for each connecting bridge (either pure random coil or random coil with 3/10 helix).

The accuracy of the “VVTAK Connecting Bridges” algorithm has been checked in a set of 50 proteins from different types of organisms (human, lancelet, nematode, plants, yeast, archaea, and viruses) excluding bacteria. Amino acid sequences of those 50 proteins demonstrate similarity levels lower than 25% according to the results of the “Decrease Redundancy” algorithm (http://web.expasy.org/decrease_redundancy/). A complete list of species and PDB access numbers can be found in the Supplementary Material “PDBIDS” file.

We estimated sensitivity and specificity levels for prediction of connecting bridges with pure coil and for those containing 3/10 helices. The lengths of 3/10 helices have been compared for BIB, HIH, BIH, and HIB regions. We also calculated the lengths of coil regions before and after 3/10 helices from those four types of supersecondary structure elements. There were 439 3/10 helices composed of 5 amino acid residues and 327 3/10 helices composed of 3 amino acid residues. Total amino acid usages in 3/10 helices, composed of 3 residues from BIB, HIH, BIH, and HIB regions, were compared with each other. The same kind of analysis was also applied to 3/10 helices composed of 5 residues. Amino acid usage has also been calculated in each position of 3/10 helices composed of 3 and 5 residues.

Detailed tables containing ratios between usage of each amino acid in the four types of connecting bridges, with and without 3/10 helices, as well as in 3/10 helices composed of 3 and 5 amino acids (in general and in each of their positions) can be found in the Supplementary Material “Ratios” file. Significant differences in that Supplementary Material file are shown in bold. The significance of all those differences has been tested via* t*-test with a threshold of* P* equal to 0.05.

We constructed a histogram representing the distribution of lengths for 4272 alpha helices from the set of proteins used in this study. The total amino acid content of N-termini (five first residues) of alpha helices has been compared with the total amino acid content of 3/10 helices composed of 5 residues. Amino acid usage has been calculated in each of the first five positions of those alpha helices and compared with amino acid usage in corresponding positions of 3/10 helices 5 residues in length (see Supplementary Material “Ratios” file).

Information entropy [19] of amino acid content distribution in 3/10 helices composed of 5 residues and of N-termini (the first five residues) of alpha helices was calculated according to the following equation:

(1) $$\begin{array}{c} H=-\sum faa\times \log_{2}faa. \end{array}$$

In (1), *H* is an information entropy (measured in bits) and *faa* is usage of a given amino acid. According to that equation, maximal entropy for a protein or its part (it is a system composed of 20 possible types of elements, that is, of 20 amino acids) is equal to 4.32 bits.

## 3. Results

### 3.1. Differences in the Amino Acid Content of Connecting Bridges between Major Secondary Structure Elements with and without 3/10 Helices

**Figure 1:**
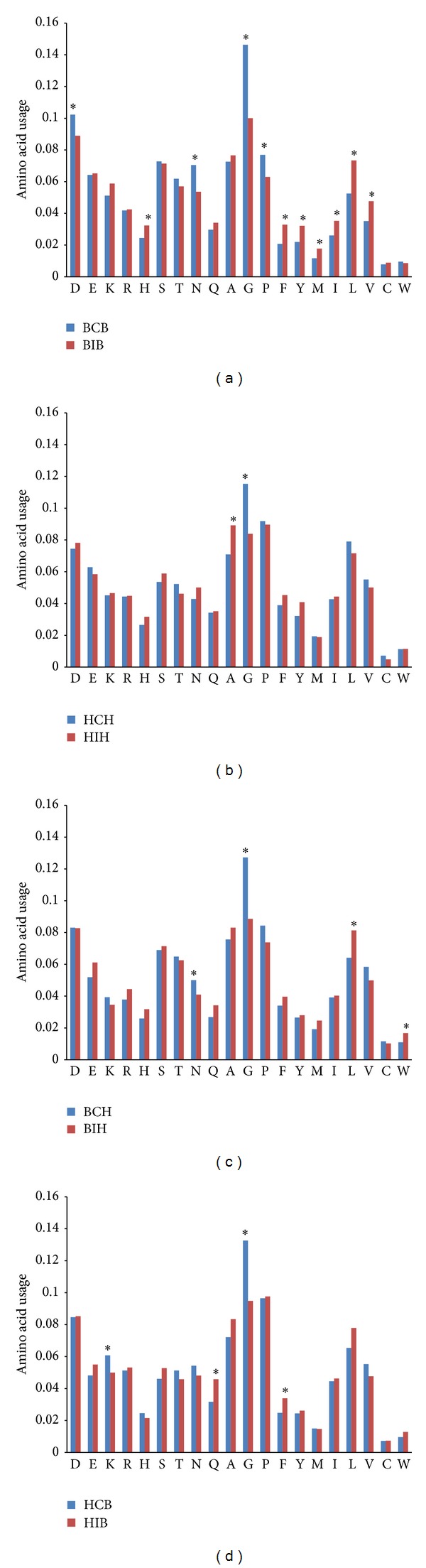
Amino acid content of “connecting bridges” with (BIB, HIH, BIH, and HIB) and without 3/10 helices (BCB, HCH, BCH, and HCB) situated between two beta strands (a), between two alpha helices (b), between beta strand and alpha helix (c), and between alpha helix and beta strand (d).

There are several differences between BCB and BIB regions (see Figure 1(a)). Four amino acid residues, known as coil formers [13] (Asp, Asn, Gly, and Pro), are used in pure random coil regions between two beta strands significantly (*P* < 0.05) more frequently than in connecting bridges containing 3/10 helices. For glycine usage the difference is especially high (it is used almost 1.5 times more frequently in BCB regions than in BIB ones). Use of His and such hydrophobic amino acids as Phe, Tyr, Met, Ile, Leu, and Val is significantly (*P* < 0.05) higher in BIB regions than in BCB regions (see Figure 1(a)). Thus we may conclude that connecting bridges between two beta strands* containing* 3/10 helices are indeed different from those connecting bridges* without* 3/10 helices.

On one hand, high usage of such helix breakers as Gly, Pro, Asp, and Asn may not allow 3/10 helix formation in BCB regions. On the other hand, the appearance (by the way of mutation) of strong beta sheet formers [13] (Phe, Tyr, Ile, and Val) in the region between two beta strands may lead to the elongation of those beta strands or to the formation of additional beta strands. In the majority of cases that kind of structural change is likely eliminated by negative selection. Structural changes will be much less drastic, for instance, if newly appeared beta sheet formers take part in 3/10 helix formation between two beta strands. That is why beta formers, as well as Met and Leu (which are also frequently found in beta strands) [18], “survive” at a higher rate in the space between two beta strands when there is a possibility for them to form 3/10 helices.

There are just two significant differences (*P* < 0.05) between the amino acid content of HCH and HIH regions (see Figure 1(b)). Glycine is used more frequently in pure coils, while alanine is used more frequently in connecting bridges containing 3/10 helices. So 3/10 helices from regions between two alpha helices have amino acid content very similar to the amino acid content of coils between alpha helices.

As to BCH and BIH regions (see Figure 1(c)), there are four significant differences (*P* < 0.05) in their amino acid content. Glycine and asparagine are more frequently found in BCH, while leucine and tryptophan are more frequently found in BIH.

In Figure 1(d), one can see that Gly and Lys are used significantly more frequently (*P* < 0.05) in HCB regions than in HIB regions, while Gln and Phe are significantly more frequently used in HIB regions.

According to our data, structural transitions from coil to 3/10 helix and vice versa should be frequent in regions connecting two alpha helices, alpha helices and beta strands, and beta strands and alpha helices. 3/10 helices in regions between two beta strands seem to be more concrete and stable.

In general, all the pure random coil regions are enriched by Gly relative to the connecting bridges containing 3/10 helices. Such strong helix-formers as Ala, Gln, and Leu [20] are found significantly more frequently only in certain types of connecting bridges containing 3/10 helices than in pure coil.

The amino acid content of coil regions strongly depends on flanking major secondary structure elements [12]. If there are 20 amino acid residues and 4 types of random coil regions (BCB, HCH, BCH, and HCB), then there are 120 pairs of amino acid usage levels that we can compare with each other (e.g., the usage of Pro in BCB and HCH, the usage of Ser in BCH and HCB, etc.). In this redundant data set, 57 of 120 (47.5%) differences are significant (see Supplementary Material “Ratios” file). As one can see in Figure 1, nearly a half of the trends existing in pure random coil regions can be seen in connecting bridges containing 3/10 helices. Indeed, 26 in 120 differences (21.7%) in amino acid usage between different types of connecting bridges containing 3/10 helices are significant (see Supplementary Material “Ratios” file).

Importantly, Gly has significantly higher (*P* < 0.05) frequency of usage in BCB and BIB regions than in HCH and HIH ones. In contrast, Pro has significantly lower (*P* < 0.05) frequency of usage in BCB and BIB regions than in HCH and HIH ones. Moreover, Pro demonstrates significantly higher (*P* < 0.05) frequencies of usage* after* alpha helices (in HCB and HIB regions) than* before *them (in BCH and BIH ones).

Usages of Ser and Thr are significantly higher (*P* < 0.05) in BCH and BIH regions than in HCB and HIB supersecondary structure elements. In contrast, the usage of Lys is significantly lower (*P* < 0.05) in BCH and BIH than in HCB and HIB regions. However, the usage of Arg that is significantly lower in BCH than in HCB regions [12] is almost equal in BIH and HIB ones. It seems like Arg has a higher probability of being incorporated into the connecting bridge between beta strand and alpha helix in case if this amino acid residue takes part in a 3/10 helix formation.

The usage of Asp is significantly higher in BCB regions than in other types of coil regions [12]. However, there are no significant differences in Asp usages between BIB, HIH, BIH, and HIB regions. It seems likely that 3/10 helices cannot incorporate multiple Asp residues. Because of this reason 3/10 helices may be formed if the usage of Asp between two beta strands is lower than a certain threshold.

### 3.2. “VVTAK Connecting Bridges” Algorithm for Testing Connecting Bridges for the Possible Presence of 3/10 Helices

The “VVTAK Connecting Bridges” algorithm was created on the basis of bacterial proteins analysis. For this reason it has been tested on a set of proteins which belong to different types of organisms, but not to bacteria. On that set made from 50 proteins which has pairwise similarity levels lower than 25%, the algorithm demonstrated 69.86% sensitivity for connecting bridges with 3/10 helices and 70.86% sensitivity for connecting bridges with pure random coil. The algorithm showed high level of specificity (93.54%) for connecting bridges made from pure random coil. Therefore, the algorithm should be accurate in predictions of connecting bridges that are unable to form a 3/10 helix. The level of specificity for connecting bridges containing 3/10 helix prediction is equal to 29.14%. This means that the algorithm overpredicts connecting bridges with 3/10 helices. However, comparisons of 3D structures of identical proteins show that the number of connecting bridges in which 3/10 helices may be formed is higher than it initially seems.

For example, in the 1V1G file from our training set, there are no 3/10 helices in connecting bridges, while the algorithm predicts that one of the regions is prone to 3/10 helix formation. On the 100% identical structure (2EHB) a short 3/10 helix (residues 101–103) is formed in that region. On the 1V1G structure, an iodide ion can be found near 101–103 residues. So one may speculate that interactions with ligand cause the destruction of 3/10 helices. Some other similar cases have been described for apo- and holo-forms of proteins coordinating Mn^2+^ ions [21]. Of course, ion binding is just one of the multiple causes of 3/10 helices disappearing. From that point of view, one can say that the “VVTAK Connecting Bridges” algorithm predicts connecting bridges which are (according to their amino acid content) prone to form 3/10 helices in certain conditions.

The aim of the “VVTAK Connecting Bridges” algorithm is not to predict 3/10 helices, but to test connecting bridges between major elements of secondary structure for their ability to form 3/10 helices. In other words, the algorithm shows whether a given connecting bridge is prone to forming 3/10 helices or whether it has no such ability. It also shows whether a given 3/10 helix is really stable. That kind of information is important for synthetic vaccine design studies. One of the examples of “VVTAK Connecting Bridges” algorithm usage is given below.

**Figure 2:**
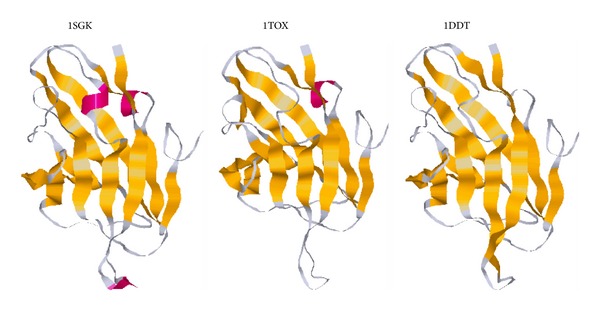
Three different 3D structures of diphtheria toxin receptor binding domain.

Three examples of 3/10 helices that seem unstable may be found in the beta-structural (receptor-binding) domain of diphtheria toxin. In the 3D structure with 1SGK PDB identifier  [22] those 3/10 helices can be found in three regions between two beta strands (see Figure 2). In the 1TOX 3D structure with a 100% identical amino acid sequence [23], there are random coil regions instead of two from three abovementioned 3/10 helices (see Figure 2). Yet another structure (1DDT) with 100% identical amino acid sequence [24] lacks all three 3/10 helices (see Figure 2). Which of the structures is more reliable? One may try to check the probability of 3/10 helix formation with the help of “VVTAK Connecting Bridges” algorithm.

In the first connecting bridge (residues 399–404), the probability of a random coil state is higher than the probability of 3/10 helix formation, while in two others (494–507 and 512–525) the state of BIB is more probable than the state of BCB.

An especially interesting example of structural variations can be observed in the 512–525 region. There is a 3/10 helix of three amino acid residues in length (518–520), which may turn to random coil. However, according to the results of “VVTAK Connecting Bridges,” the probability for 3/10 helix formation is higher than the probability of pure coil existence when the length of the corresponding connecting bridge is long (512–525 in 1SGK and 1TOX structures) and short (517–522 in 1DDT structure). The entire beta hairpin (508–530) is the less mutable epitope of diphtheria toxin, which was suggested by us for synthetic vaccine development [25]. So if the probability of regular structure formation on the top of its loop is higher than the probability of random coil existence, it may be yet another benefit of its usage in peptide vaccine studies. Experiments showed that the synthetic peptide corresponding to that beta hairpin (the SF23 peptide) reproduced the structure of the epitope of the full-length diphtheria toxin [26].

### 3.3. Typical Length of 3/10 Helices Depends on Flanking Elements of Secondary Structure

**Figure 3:**
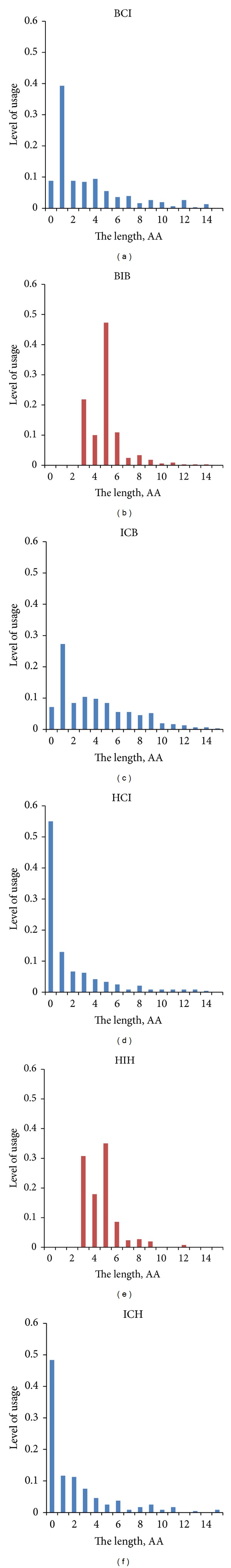
Lengths of 3/10 helices (b, e) and flanking coil regions (a, c, d, f) situated between two beta strands (a, b, c) and between two alpha helices (d, e, f). For connecting bridges between two beta strands: BCI is a region of coil between beta strand and 3/10 helix; BIB is a 3/10 helix between two beta strands; ICB is a region of coil between 3/10 helix and beta strand. For connecting bridges between two alpha helices: HCI is a region of coil between alpha helix and 3/10 helix; HIH is a 3/10 helix between two alpha helices; ICH is a region of coil between 3/10 helix and alpha helix.

**Figure 4:**
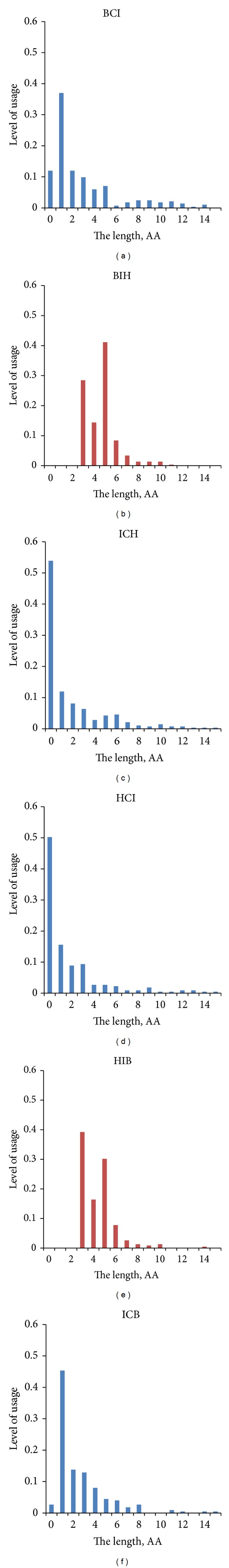
Lengths of 3/10 helices (b, e) and flanking coil regions (a, c, d, f) situated between beta strand and alpha helix (a, b, c) and between alpha helix and beta strand (d, e, f). For connecting bridges between beta strands and alpha helices: BCI is a region of coil between beta strand and 3/10 helix; BIH is a 3/10 helix between beta strand and alpha helix; ICH is a region of coil between 3/10 helix and alpha helix. For connecting bridges between alpha helices and beta strands: HCI is a region of coil between alpha helix and 3/10 helix; HIB is a 3/10 helix between alpha helix and beta strand; ICB is a region of coil between 3/10 helix and beta strand.

In general, there are two most common lengths of 3/10 helices: 3 and 5 amino acid residues (see Figures 3 and 4). There are just two hydrogen bonds (two consecutive turns) in 3/10 helices composed of 3 amino acids [10]. 3/10 helices composed of 5 residues should have four hydrogen bonds (four consecutive turns). Interestingly, 3/10 helices composed of 4 and 6 amino acids are less frequent than those composed of 3 and 5 residues. 3/10 helices longer than 6 residues in length are extremely rare. According to our results, the typical length of 3/10 helices strongly depends on the flanking elements of secondary structure.

The most common length of a 3/10 helix situated between two beta strands is equal to 5 amino acid residues (see Figure 3(b)). Almost a half (47.27%) of 3/10 helices situated in BIB elements of supersecondary structure are composed of 5 amino acid residues. The percentage of 3/10 helices composed of 5 amino acids is significantly higher (*P* < 0.05) in BIB elements than the percentage of 3/10 helices composed of 3 amino acids (21.82%), as illustrated in Figure 3(b).

Even though the percentage of 3/10 helices composed of 5 amino acids (35.02%) is higher than the percentage of 3/10 helices composed of 3 amino acids (30.74%) in HIH elements of supersecondary structure, the difference between those percentages is insignificant. In other words, 3/10 helices composed of 3 and 5 amino acid residues are equivalently frequent in regions between two alpha helices (see Figure 3(e)).

In BIB regions 3/10 helices are usually separated from both beta strands by a single amino acid residue (see Figures 3(a) and 3(c)). In the case of HIH regions, 3/10 helices are usually not separated from C-terminal amino acid residues of the first and N-terminal residues of the second alpha helix (see Figures 3(d) and 3(f)).

The difference between usages of 3/10 helices 5 amino acids in length (41.14%) and 3 amino acids in length (28.43%) is significant (*P* < 0.05) for BIH regions of supersecondary structure (see Figure 4(b)). In contrast (see Figure 4(e)), 3/10 helices of the shortest possible length are significantly (*P* < 0.05) more frequent (39.22%) in HIB regions of supersecondary structure than 3/10 helices composed of 5 amino acids (30.17%).

According to the data from Figures 3 and 4, those 3/10 helices which are situated after the alpha helix (in HIB regions) usually have a length of 3 amino acids, while 3/10 helices situated before the alpha helix (in BIH regions) are usually composed of 5 amino acids. Since 3/10 helices in HIH regions consist of those situated both after and before alpha helices, they demonstrate two typical lengths (3 and 5 residues).

As one can see in both Figures 3 and 4, 3/10 helices are usually separated by a single amino acid residue (whose structural state is described as random coil) from beta strands, and they are usually not separated by random coil from alpha helices.

### 3.4. Amino Acid Content of 3/10 Helices 3 Amino Acids in Length Shows Stronger Dependence on the Flanking Elements of Secondary Structure Than That of 3/10 Helices 5 Amino Acids in Length

**Figure 5:**
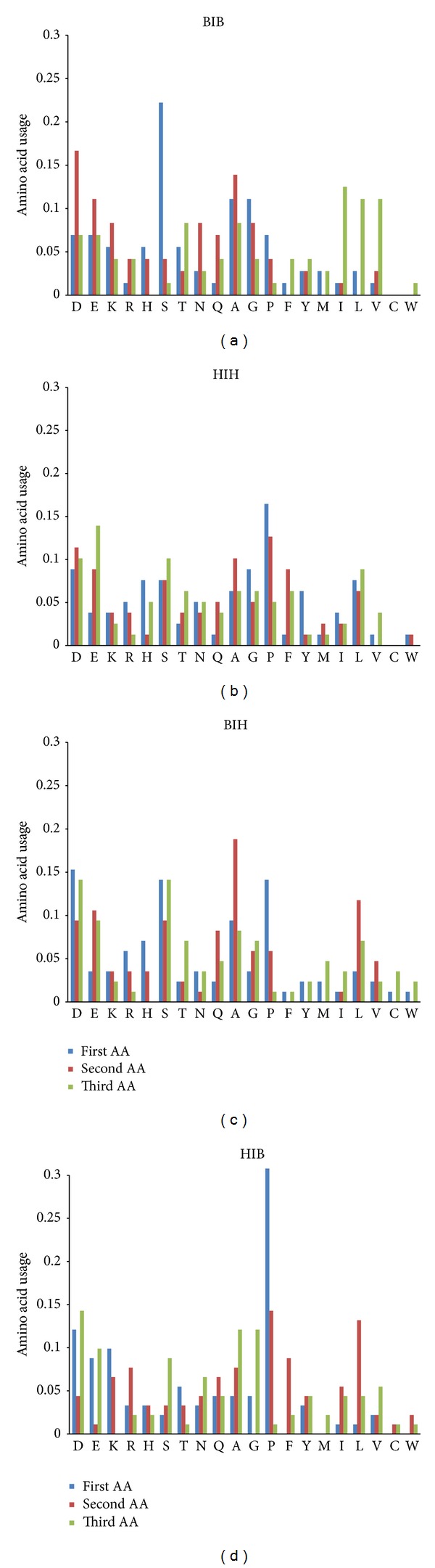
Position-specific amino acid content of 3/10 helices composed of 3 residues situated between two beta strands (a), between two alpha helices (b), between beta strand and alpha helix (c) and between alpha helix and beta strand (d).

In Figure 5 we have shown amino acid content in every position for 3/10 helices composed of 3 amino acid residues. As one can see, amino acid composition of the shortest 3/10 helices strongly depends on flanking elements of secondary structure.

Interestingly, (see Figure 5(a)) 3/10 helices situated between two beta strands preferably have Ser in their first positions. Hydrophobic Ile, Val, and Leu (strong formers of beta strands [13]) are found preferably in third positions.

In the space between two alpha helices, position-specific preferences for the shortest 3/10 helices are different (see Figure 5(b)). First positions, as well as second positions, are typically occupied by Pro.

3/10 helices composed of three amino acid residues situated in BIH regions can be described in a different way (see Figure 5(c)). First positions preferably contain such coil makers [13], as Asp, Ser, and Pro, while second positions are enriched by Ala (strong helix former [13]). In general, Ser usage is significantly higher (*P* < 0.05) in the shortest 3/10 helices from BIH than in those from HIB regions. The general usage of Pro in 3/10 helices from BIH is significantly lower (*P* < 0.05) than in 3/10 helices from HIB.

The shortest 3/10 helices from HIB regions have extremely high use of Pro as their first amino acid (see Figure 5(d)). It is interesting to highlight that Lys also has a high frequency of usage in first positions of 3/10 helices from HIB.

Some trends in amino acid content distribution between four types of 3/10 helices composed of 3 amino acid residues resemble the trends found in the four types of random coil regions (see Supplementary Material “Ratios” file). Alanine is used significantly more frequently in shortest 3/10 helices from BIH than in those from HIH and HIB. Leucine appears more frequently in 3/10 helices from BIH and HIH than in those from BIB. Extremely high usage of Pro is a characteristic of shortest 3/10 helices from both HIH and HIB regions (in their first and second positions). Ser makes up the highest level of usage in 3/10 helices from BIH regions (namely, in their first and third positions), while the lowest level of Ser usage is a characteristic of 3/10 helices from HIB regions.

Some other trends should also be mentioned. Lysine has significantly higher frequency of usage in 3/10 helices from BIB than in those from HIH and BIH. 3/10 helices from BIB are also significantly enriched by Asn, by Ile, and by Tyr (relative to those from BIH), as well as by Val (relative to those from HIH).

### 3.5. Amino Acid Content of 3/10 Helices 5 Amino Acids in Length Shows Weaker Dependence on the Flanking Elements of Secondary Structure Than That of 3/10 Helices 3 Amino Acids in Length

Total amino acid usage in 3/10 helices 5 residues in length is almost the same for 3/10 helices from BIB, HIH, BIH, and HIB regions. Here we should say that, for 3/10 helices composed of 3 residues from four types of connecting bridges, the number of significant differences in amino acid use is equal to 28 from 120 (23.3%). For 3/10 helices composed of five residues there are just 3 from 120 (2.5%) significant differences (see Supplementary Material “Ratios” file). Firstly, Pro is used significantly more frequently in 3/10 helices from HIH and HIB regions than in those from BIB regions. Secondly, Gly is used significantly more frequently in 3/10 helices from BIB than in those from HIH regions. Some other significant trends can be found only in certain amino acid positions.

**Figure 6:**
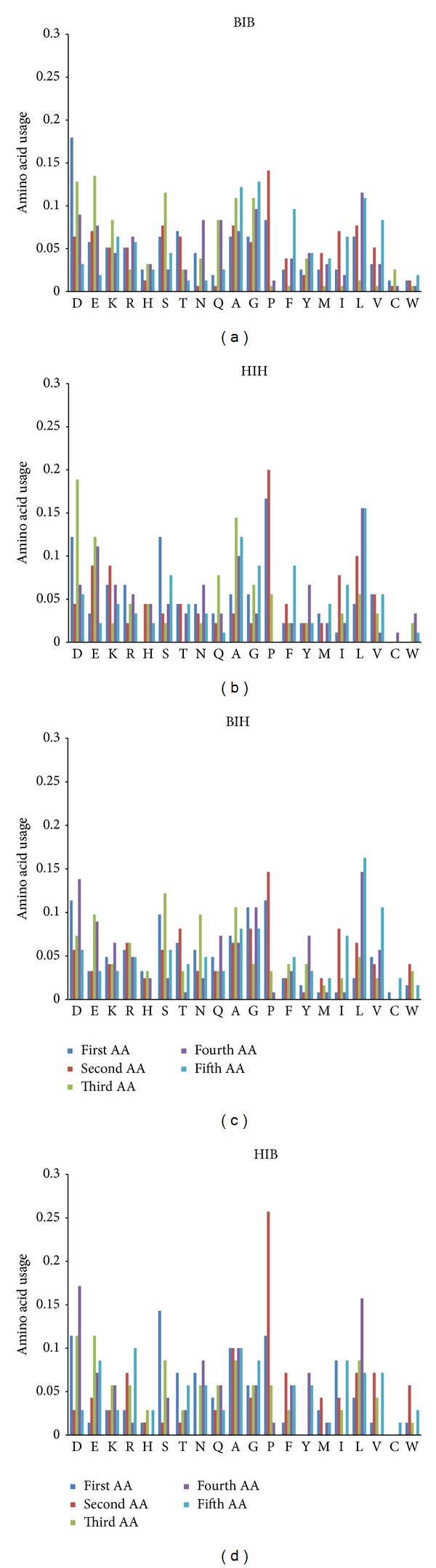
Position-specific amino acid content of 3/10 helices composed of 5 residues situated between two beta strands (a), between two alpha helices (b), between beta strand and alpha helix (c), and between alpha helix and beta strand (d).

Usages of Ser and Thr are significantly higher in the second position of 3/10 helices from BIB than in that from HIB (Figure 6). Usage of Thr is significantly higher when in the second position of 3/10 helices from BIH than in that from HIB regions. Use of Ser and Thr is significantly higher in the third position of 3/10 helices from BIB and BIH than in that from HIH regions. Use of Ser is significantly higher in the fifth position in 3/10 helices from BIB and BIH than in that from HIB. Those trends follow the distribution of Ser and Thr between different types of coil: BCB and BCH regions have higher levels of those amino acids than HCB regions [12]. It is interesting to note that trends described for Ser and Thr distribution are not repeated by Tyr distribution, even though it also possesses –OH group on its side chain (see Supplementary Material “Ratios” file). Probably, relatively hydrophilic Ser and Thr side chains serve as stabilizers of beta strands' C-termini being situated in random coil after beta strands, while hydrophobic Tyr residues are more frequently included directly in beta-strands [18].

In general, both the total amino acid content and the distribution of amino acids between positions have much in common for 3/10 helices 5 residues in length from all the four types of connecting bridges (see Figure 6). Asp demonstrates elevated level of usage not only in first position but also in third and fourth positions (in all the four types of regions). Usage of Glu is also elevated in third and fourth positions. Such hydrophobic amino acids as Leu, Val, Ile, and Phe usually demonstrate elevated levels of usage in second, fourth, and fifth positions. Moreover, Leu more frequently occupies fourth positions, while Phe, Ile, and Val have especially high levels in fifth positions. Those position-specific trends in amino acid usage described above for 3/10 helices composed of 5 residues resemble known preferences for N-termini of alpha helices [8, 14].

## 4. Discussion

In this work we revisited the question about the nature of 3/10 helices. Do they more resemble alpha helices or regions of coil? Amino acid content of coil regions strongly depends on their location [12]. Coils between two beta strands are different from the coils between two alpha helices; the coil between beta strands and alpha helices also differs from the coil between alpha helices and beta strands. For this reason, there was the only one way to answer the question about the nature of 3/10 helices: to compare “bridges” connecting different elements of secondary structure including 3/10 helices and regions of coil without 3/10 helices.

We found that 3/10 helices situated between two beta strands have more features distinguishing them from the random coil than 3/10 helices situated between other major secondary structure elements. If 3/10 helices situated between beta strands show more differences from the corresponding coil, they should have more stable and defined structures. Antigenic epitopes composed of 3/10 helices situated in loops between two beta strands should be more structurally stable than those composed of 3/10 helices situated in other elements of supersecondary structure. Moreover, 3/10 helices in BIB regions usually include hydrophobic amino acids (Val, Ile, Leu, and Phe), which are thought to be highly antigenic if they are situated on the surface of a protein [27]. We have previously described an example of such an epitope from the diphtheria toxin [25]. In this work we confirmed that beta hairpin from the less mutable epitope of diphtheria toxin has a 3/10 helix on its top at a higher probability than random coil. Such tests have been performed by the “VVTAK Connecting Bridges” algorithm (http://chemres.bsmu.by/) that works on the basis of propensity scales created after the analysis of amino acid usage in connecting bridges with and without 3/10 helices.

**Figure 7:**
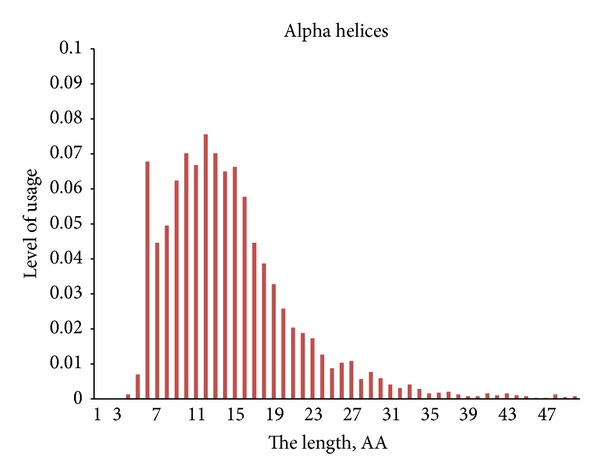
Lengths of alpha helices.

There are two most common lengths of 3/10 helices: 3 residues and 5 residues. Amino acids composed of 3/10 helices from 3 residues show stronger dependence on flanking elements of secondary structure than amino acid content of 3/10 helices formed from 5 residues. Therefore we can say that 3/10 helices formed by 3 amino acids resemble fragments of random coil, while 3/10 helices from 5 amino acids resemble N-termini of alpha helices. As shown in Figure 7, alpha helices composed of 4 and 5 amino acids are very rare. It seems like helices composed of 4 and especially of 5 residues are mostly 3/10 and not alpha helices.

**Figure 8:**
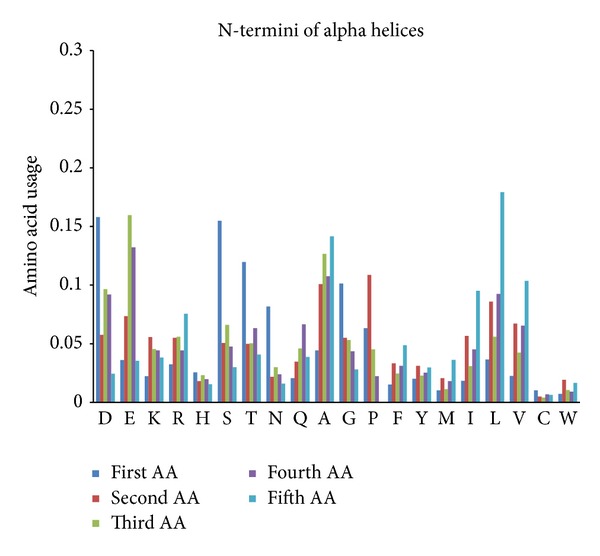
Position-specific amino acid content of N-termini (the first five positions) of alpha helices.

One may say that 3/10 helices made from 5 amino acids are just short versions of alpha helices with different hydrogen bonding, but this is not exactly true. Even though position-specific amino acid propensities of 3/10 helices composed of 5 residues resemble those of five N-terminal amino acid positions of alpha helices, there are still some differences between them. For example, proline is the most frequently used amino acid in second positions of alpha helices (see Figure 8), while its usage in those positions of 3/10 helices composed of 5 residues is significantly higher (as well as in first positions). In contrast, N-termini of alpha helices have significantly higher levels of Glu, Thr, Ala, and Val (see Supplementary Material “Ratios” file).

In general, N-termini of alpha helices seem to have stronger position-specific amino acid preferences (and better defined structures as a result) than 3/10 helices composed of 5 residues. Indeed, the informational entropy is some lower for amino acid content distribution of alpha helices N-termini (4.0999 bits) than for 3/10 helices from 5 residues (4.1471 bits).

One has to remember that N-termini of alpha helices are enriched by coil formers (by Asp, Ser, Pro, and Asn) [14]. So, 3/10 helices composed of 5 residues resemble N-termini of alpha helices, while N-termini of alpha helices themselves resemble random coil. Thus we may expect that some 3/10 helices formed from 5 residues are remains of N-termini of alpha helices (“cores” of those alpha helices might be destroyed by mutations, or, alternatively, nucleotide sequences encoding N-termini of alpha helices might be duplicated and translocated to other parts of the coding region).

Functional groups able to bind ligands (including ions) should be already connected by hydrogen bonds (“side chain-side chain” or “side chain-main chain” ones) or involved in polar interactions with other amino acids more frequently if they are included in 3/10 helices than if they are situated in pure random coil [28]. On one hand, amino acids from 3/10 helices should bind ligands less effectively than those from coil (as they actually do [21]). On the other hand, 3/10 helices may sometimes disappear after the binding of ion or other ligand due to the destruction of functional group interactions stabilizing those 3/10 helices. Even small changes in angles and distances between N–H and C=O groups from the protein backbone may lead to situations where DSSP program no longer recognizes “main chain-main chain” hydrogen bond.

What are the factors that influence the length of 3/10 helices? What causes the decrease of their lengths in HIH and especially in HIB regions? Let us consider that 3/10 helix can occasionally be formed from random coil due to a single amino acid replacement. In that case, its length may be influenced by such a strong helix breaker as proline [13, 29]. One proline residue can be situated in the first and especially in the second position of a 3/10 helix, while the second proline residue should break that 3/10 helix. Sufficient space between two Pro residues in coil may be important for 3/10 helix formation and for its final length. According to our calculations, about 32% of coil regions have at least one Pro residue. This statement is true for all of the four types of coil: BCB, HCH, BCH, and HCB. However, the usage of Pro is significantly higher in HCH and HCB regions than in BCB and BCH ones [12]. How can this be explained? In HCH and HCB two or more Pro residues can be found more frequently than in BCB and BCH ones. Moreover, according to our calculations, Pro-Xaa-Pro motifs are used in HCH and HCB regions more frequently than Pro-Xaa-Xaa-Pro motifs. In contrast, Pro-Xaa-Xaa-Pro motifs are more abundant in BCB and BCH regions than Pro-Xaa-Pro ones. Thus the length of the available space for 3/10 helix formation is longer in BCB and BCH regions than in HCH and HCB ones.

## 5. Conclusions

Approximately every seventh connecting bridge between major secondary structure elements (alpha helices and beta strands) includes a 3/10 helix. All four types of connecting bridges containing 3/10 helices have significantly lower usage of glycine than those composed of “pure” coil.

Hydrophobic amino acids are more frequently incorporated into 3/10 helices (in third positions of helices 3 residues in length and in fourth and fifth positions of helices 5 residues in length) situated between two beta strands, rather than into the pure random coil situated between two beta strands.

The usage of proline is higher in connecting bridges situated after alpha helices than in those situated after beta strands or between two beta strands. This statement is true for pure coil, 3/10 helices composed of 3 amino acids, and 3/10 helices composed of 5 amino acid residues. Elevated usage of Pro may be one of the factors responsible for the short typical length (3 residues) of 3/10 helices in regions between alpha helix and beta strand.

Amino acid content of 3/10 helices composed of 3 residues shows more significant dependences on flanking elements of secondary structure than amino acid content of 3/10 helices composed of 5 residues does.

## Supplementary Material

“PDBIDS” file includes PDB identifiers for proteins from each bacteria used in the main data set, as well as PDB indentifiers for nonbacterial proteins from the training set. “Ratios” file includes tables with ratios between amino acid usages in four types of connecting bridges (for “pure” random coil, for coil with 3/10 helices, for 3/10 helices 3 and 5 residues in length), as well as between amino acid usage in first five positions of alpha helices and in 3/10 helices 5 amino acid residues in length.

## Abbreviations

BIB: Connecting bridge between two beta strands containing 3/10 helix
BCB: Connecting bridge between two beta strands without 3/10 helix
HIH: Connecting bridge between two alpha helices containing 3/10 helix
HCH: Connecting bridge between two alpha helices without 3/10 helix
BIH: Connecting bridge between beta strand and alpha helix containing 3/10 helix
BCH: Connecting bridge between beta strand and alpha helix without 3/10 helix
HIB: Connecting bridge between alpha helix and beta strand containing 3/10 helix
HCB: Connecting bridge between alpha helix and beta strand without 3/10 helix.
